# Factors Associated with Clinically Inadequate Bowel Preparation Among Patients with Diabetes Mellitus Undergoing Colonoscopy: A Single-Center Observational Study

**DOI:** 10.3390/diagnostics16182935

**Published:** 2026-09-11

**Authors:** Ivana Jukić, Duje Denona, Tina Bečić, Ajka Pribisalić, Josipa Radić, Mislav Radić, Damir Fabijanić, Vedran Kovačić, Jonatan Vuković

**Affiliations:** 1 Division of Gastroenterology, Department of Internal Medicine, University Hospital of Split, 21000 Split, Croatia; ivjukic@gmail.com (I.J.); jonatan.vukovic@mefst.hr (J.V.); 2 Faculty of Health Sciences, University of Split, 21000 Split, Croatia; 3 School of Medicine, University of Split, 21000 Split, Croatia; duje.denona13@gmail.com; 4 Department of Cardiovascular Diseases, University Hospital of Split, 21000 Split, Croatia; damirfabijanic62@gmail.com; 5 Division of Nephrology, Dialysis and Arterial Hypertension, Department of Internal Medicine, University Hospital of Split, 21000 Split, Croatia; josiparadic1973@gmail.com; 6 Department of Internal Medicine, School of Medicine, University of Split, 21000 Split, Croatia; mislavradic@gmail.com (M.R.); vedran.kovacic.split@gmail.com (V.K.); 7 Division of Rheumatology, Allergology and Clinical Immunology, Department of Internal Medicine, University Hospital of Split, 21000 Split, Croatia; 8 Department of Clinical Propedeutics, School of Medicine, University of Split, 21000 Split, Croatia; 9 Division of Internistic Intensive Medicine with Clinical Pharmacology and Toxicology, Department of Internal Medicine, School of Medicine, University Hospital of Split, University of Split, 21000 Split, Croatia

**Keywords:** diabetes mellitus, colonoscopy, bowel preparation, bowel cleansing, Boston Bowel Preparation Scale, diabetes duration, inadequate bowel preparation

## Abstract

**Background**: Adequate bowel preparation is essential for high-quality colonoscopy, yet patients with diabetes mellitus remain particularly vulnerable to suboptimal cleansing. This study evaluated factors associated with clinically inadequate cleansing among patients with diabetes mellitus undergoing colonoscopy. **Methods:** This ambispective observational study included 194 adult patients with diabetes mellitus who underwent total colonoscopy at University Hospital Center Split between 1 January 2019 and 30 October 2025 and were prepared using an institutional split-dose PEG/macrogol-based bowel preparation approach. Bowel preparation quality was assessed using the Boston Bowel Preparation Scale (BBPS). Clinically inadequate bowel preparation was defined as total BBPS ≤ 5 or at least one segmental BBPS score ≤ 1. Multivariable logistic regression was used to evaluate associated factors. **Results:** Median age was 69 years, 131 participants (67.5%) were male, and 179 (92.3%) had type 2 diabetes mellitus. Clinically inadequate bowel preparation was observed in 68/194 participants (35.1%; 95% CI: 28.3–41.8%). In the main complete-case model (*N* = 147; events = 52), longer diabetes duration remained associated with clinically inadequate preparation after multivariable adjustment. Each 5-year increase in diabetes duration was associated with higher odds after adjustment for age, sex, outpatient status, chronic constipation, and use of tricyclic antidepressants or opioids (adjusted OR 1.27, 95% CI: 1.05–1.54; *p* = 0.015). Sensitivity analyses supported this association. **Conclusions:** Clinically inadequate bowel preparation was frequent despite the institutional split-dose PEG/macrogol-based bowel preparation approach. Longer diabetes duration may represent a candidate clinical variable for future risk-stratification studies, but it should not be used as a stand-alone criterion for intensified bowel preparation without prospective validation.

## 1. Introduction

Colonoscopy remains the cornerstone diagnostic and therapeutic procedure for the detection, surveillance, and prevention of colorectal cancer, as well as for the evaluation of a broad spectrum of gastrointestinal disorders. Its diagnostic accuracy is highly dependent on the quality of bowel preparation, since inadequate cleansing may impair mucosal visualization, reduce adenoma detection rates, prolong procedure duration, increase patient discomfort, and lead to repeat examinations [1,2,3]. Therefore, optimization of bowel preparation represents a key quality indicator in colonoscopy practice [4].

Despite continuous improvements in bowel cleansing regimens, inadequate bowel preparation remains a common clinical problem, particularly among patients with risk factors for impaired gastrointestinal motility. Diabetes mellitus is one of the most frequently reported predictors of suboptimal bowel preparation [5,6,7,8,9,10]. Several mechanisms may explain this association, including delayed gastric emptying, autonomic neuropathy, altered colonic transit, chronic constipation, fluid and electrolyte disturbances and the complexity of periprocedural adjustment of antihyperglycemic therapy [10,11,12]. These factors may be especially relevant in patients with long-standing diabetes and diabetes-related complications.

Patients with diabetes mellitus may experience impaired bowel cleansing through both direct and indirect mechanisms. Chronic hyperglycemia and diabetes-related autonomic dysfunction may alter gastrointestinal motility, while diabetic gastroparesis and delayed colonic transit can reduce the effectiveness of standard laxative regimens. In addition, dietary restrictions before colonoscopy and fasting during bowel preparation may destabilize glycemic control, increasing the risk of hypoglycemia or hyperglycemia. The need to adjust insulin and other antihyperglycemic medications further complicates preparation in this population, particularly in older patients and those with multiple comorbidities [11,12].

The importance of this issue is further emphasized by the increasing global burden of both diabetes mellitus and colorectal cancer. Patients with diabetes represent a growing population undergoing colonoscopy for screening, surveillance, and diagnostic indications. At the same time, current bowel preparation guidelines provide limited diabetic-specific recommendations for this high-risk group [13,14,15]. A recent review specifically addressing bowel preparation for colonoscopy in patients with diabetes mellitus emphasized that diabetes is highly likely associated with inadequate bowel cleansing and highlighted the lack of detailed diabetes-specific recommendations in major gastroenterology guidelines [5]. Although general recommendations emphasize split-dose preparation, adequate patient education, and appropriate timing of bowel cleansing agents, they do not sufficiently address diabetes-specific challenges such as glucose monitoring, medication adjustment, gastrointestinal dysmotility, renal impairment or the use of newer antihyperglycemic agents. This evidence gap supports the need for real-world studies evaluating bowel preparation quality and associated risk factors within diabetic populations.

Previous studies and meta-analyses have consistently shown that diabetes mellitus is associated with a higher risk of inadequate bowel cleansing [6,7,8,9,10]. However, available evidence remains heterogeneous, and many studies include diabetic patients only as a subgroup within broader cohorts. In addition, relatively few studies have focused exclusively on diabetic patients and evaluated clinical, metabolic, comorbidity-related, and laboratory factors associated with inadequate bowel preparation in real-world practice. Consequently, there is still a need for additional data that may help identify diabetic patients at particularly high risk for poor bowel cleansing and guide individualized preparation strategies.

The Boston Bowel Preparation Scale (BBPS) is a validated and widely used scoring system for assessing bowel cleanliness during colonoscopy [16,17]. It evaluates three colonic segments and provides a total score ranging from 0 to 9, allowing standardized assessment of preparation quality. In clinical practice and research, a total BBPS score of ≥6 is commonly considered adequate, whereas lower values indicate insufficient preparation. However, a total-score definition alone may classify some patients as adequately prepared despite inadequate cleansing in an individual colonic segment. Therefore, incorporating segmental BBPS scores may provide a more clinically relevant assessment of bowel preparation adequacy. The use of a standardized scale such as the BBPS allows more objective comparison of bowel preparation quality and facilitates identification of patients with inadequate cleansing.

The aim of the present ambispective observational study was to evaluate bowel preparation quality in adult patients with diabetes mellitus undergoing total colonoscopy using an institutional split-dose PEG/macrogol-based bowel preparation approach.

In addition, we aimed to assess the frequency of clinically inadequate bowel preparation, defined as a total BBPS score ≤ 5 or at least one segmental BBPS score ≤ 1, and to identify clinically accessible demographic, clinical, diabetes-related, comorbidity-related, and laboratory factors associated with inadequate bowel cleansing within a diabetic cohort. Importantly, the study was not designed to re-establish diabetes mellitus itself as a risk factor for inadequate bowel preparation, but rather to explore risk stratification among patients already diagnosed with diabetes mellitus in routine clinical practice.

## 2. Materials and Methods

### 2.1. Study Design and Participants

An ambispective observational study was conducted among adult patients with diabetes mellitus who underwent total colonoscopy at the Department of Gastroenterology and Hepatology, University Hospital Center Split. Data were collected for the period from 1 January 2019 to 30 October 2025. For patients examined from 1 January 2019 to 31 January 2025, data were collected retrospectively from electronic medical records. For patients examined from 1 February 2025 to 30 October 2025, data were collected prospectively according to the same predefined study protocol. All consecutive adult patients with diabetes mellitus who underwent total colonoscopy during the study period were identified and screened for eligibility according to the predefined inclusion and exclusion criteria.

Patients meeting the following inclusion criteria were included: age ≥ 18 years, confirmed diagnosis of diabetes mellitus (type 1 or type 2), bowel preparation performed according to the institutional split-dose polyethylene glycol (PEG)/macrogol-based bowel preparation approach and completion of total colonoscopy without anesthesia.

Exclusion criteria were acute gastrointestinal bleeding requiring a non-standard bowel preparation protocol, bowel preparation with more than two doses of polyethylene glycol, incomplete colonoscopy, age < 18 years, suspected gastrointestinal obstruction, colonoscopy performed under anesthesia, or any extended or otherwise non-standard bowel preparation protocol.

Following screening and application of the predefined eligibility criteria, three patients were excluded because only subtotal colonoscopy was achieved. In all three cases, the procedure was discontinued because of patient discomfort or pain rather than inadequate bowel preparation. A further six patients were excluded because they underwent an extended or otherwise non-standard bowel preparation protocol. The number of procedures excluded because colonoscopy was performed under anesthesia was not recorded during initial eligibility screening and therefore cannot be reliably reconstructed from the study dataset.

### 2.2. Data Collection

Data were collected from electronic medical records and included demographic characteristics (age, sex, body mass index), clinical data (type and duration of diabetes mellitus, antidiabetic therapy, comorbidities, diabetes-related complications, and relevant medical history), laboratory findings, as well as colonoscopy- and bowel preparation-related data. Colonoscopy-related variables included indication for colonoscopy, outpatient or inpatient status, previous colonoscopy, previous abdominal surgery, chronic constipation, use of tricyclic antidepressants or opioids, and acute infection at the time of colonoscopy. Chronic constipation was ascertained pragmatically based on documented clinical history in the medical records and/or colonoscopy referral. Standardized diagnostic criteria, such as the Rome IV criteria, were not systematically applied.

Comorbidities were categorized according to organ systems and included cardiovascular, nephrological, psychiatric, musculoskeletal, neurological/ophthalmological, gastrointestinal, pulmonary, endocrine, rheumatological, hematological, oncological, and urological/gynecological diseases. Diabetes-related complications were classified as microvascular (neuropathy, nephropathy, retinopathy) and macrovascular complications (cardiovascular complications, cerebrovascular complications, and peripheral vascular disease). Neuropathy was recorded as documented in the medical records; diabetic autonomic neuropathy was not separately or systematically assessed, and objective autonomic function testing was not performed.

Laboratory variables were analyzed as available and were not imputed. Because laboratory parameters were not the primary focus of the study, they were presented descriptively and included in supplementary analyses.

### 2.3. Assessment of Bowel Preparation Quality

According to the institutional protocol and the respective manufacturer’s instructions, patients underwent an institutional split-dose PEG/macrogol-based bowel preparation approach. PEG/macrogol-based preparations available at the institution were used in routine clinical practice according to the hospital protocol and the respective manufacturer’s instructions. The intended regimen consisted of two separate doses, with the first administered on the day before colonoscopy and the second on the day of the procedure. Patients were instructed to follow a low-residue diet before colonoscopy and to maintain adequate clear-fluid intake during preparation. Written instructions were provided using the center’s pre-printed bowel preparation form, and the preparation procedure was additionally explained verbally by medical staff involved in the endoscopy preparation process.

Because part of the cohort was collected retrospectively and bowel preparation was administered as part of routine clinical care, the exact commercial formulation used, the prescribed total and split-dose volumes, the volume actually consumed, the exact timing of dose completion, adherence to dietary and hydration instructions, and the precise interval between completion of the final dose and colonoscopy could not be reliably verified for all participants. Therefore, the protocol described above reflects the intended institutional split-dose PEG/macrogol-based bowel preparation approach, based on the respective manufacturer’s instructions and hospital protocol, rather than confirmed patient-level adherence to each component of the regimen.

The quality of bowel preparation was assessed using the BBPS. This scale evaluates bowel cleanliness in each of the three colonic segments: the right colon (BBPS-RC), transverse colon (BBPS-TC), and left colon/sigmoid colon (BBPS-LC). Each segment is scored on a scale from 0 to 3, where 0 indicates an unprepared colon segment with mucosa not visualized due to solid stool, and 3 indicates excellent preparation with complete visualization of the mucosa and no residual stool or liquid. The total BBPS score represents the sum of the scores from all three segments and ranges from 0 to 9.

BBPS scoring was performed by the endoscopist who conducted the colonoscopy and was documented in the colonoscopy report as part of routine clinical practice. Endoscopists were not formally blinded to patients’ clinical information when assigning BBPS scores. Nevertheless, BBPS scoring was based on direct endoscopic visualization using standardized segmental criteria, and diabetes duration is not an element of the BBPS scoring system. For the purposes of this study, segmental and total BBPS scores were extracted from the colonoscopy reports by the research team.

The primary study outcome was clinically inadequate bowel preparation, defined as a total BBPS score ≤ 5 or at least one segmental BBPS score ≤ 1. Clinically adequate bowel preparation was defined as a total BBPS score ≥ 6 with all segmental BBPS scores ≥ 2. Based on this definition, a binary outcome variable was created (0 = clinically adequate bowel preparation; 1 = clinically inadequate bowel preparation). The total-score definition of inadequate bowel preparation, defined as total BBPS ≤ 5, was used only in sensitivity analysis.

### 2.4. Statistical Analysis

Statistical analysis was performed using IBM SPSS Statistics software, version 25. The normality of distribution for continuous variables was assessed using the Shapiro–Wilk test. As most continuous variables showed statistically significant deviation from normal distribution, they were presented as medians and interquartile ranges (IQRs). Categorical variables were expressed as absolute frequencies and percentages.

Comparisons of continuous variables between patients with clinically adequate and clinically inadequate bowel preparation were performed using the Mann–Whitney U test. Comparisons of categorical variables between groups were conducted using Pearson’s chi-square test, while Fisher’s exact test was applied when the expected frequency in any cell was less than 5. Because multiple bivariate comparisons were performed, these analyses were considered exploratory. Benjamini–Hochberg false-discovery-rate (FDR) correction was applied to assess the robustness of nominally significant bivariate findings.

For bivariate analyses, individual variables were analyzed using available data only, without imputation of missing values. Multivariable logistic regression models were based on complete-case analysis. Multiple imputation was not performed because the study was exploratory, the sample size was modest, and several variables had non-negligible missingness. In addition, the available dataset did not include sufficient auxiliary variables to confidently support a robust imputation model for all clinically relevant missing data mechanisms. Therefore, the potential impact of missing data was addressed in the interpretation of the results and in the Section 4.4.

To assess potential selection related to missing diabetes-duration data, participants included in the main complete-case regression model were compared with those excluded because of missing diabetes-duration data using available baseline characteristics and the primary bowel-preparation outcome.

The prevalence of clinically inadequate bowel preparation was calculated as a proportion with a 95% confidence interval (95% CI), estimated using the normal approximation method.

To address potential confounding, multivariable logistic regression analyses were performed with clinically inadequate bowel preparation as the dependent variable. Clinically inadequate bowel preparation was defined as a total BBPS score ≤ 5 or at least one segmental BBPS score ≤ 1. Diabetes duration was entered as the main explanatory variable and expressed per 5-year increase to improve clinical interpretability. The main adjusted model included age, sex, outpatient versus inpatient status, chronic constipation, and use of tricyclic antidepressants or opioids. Covariates were selected a priori based on clinical relevance, previously reported associations with bowel preparation quality, and the need to avoid overfitting given the number of clinically inadequate bowel preparation events. Diabetes duration was the primary exposure of interest. Age and sex were included as standard demographic covariates. Outpatient status was included to account for differences in the clinical setting of colonoscopy preparation. Chronic constipation and use of tricyclic antidepressants or opioids were included because of their potential effects on bowel motility and bowel cleansing quality.

Model fit was assessed using the Hosmer–Lemeshow goodness-of-fit test, and multicollinearity was evaluated using variance inflation factors. The linearity-in-the-logit assumption for the continuous predictors age and diabetes duration was assessed using Box–Tidwell interaction terms.

Sensitivity analyses were performed to evaluate the robustness of the main finding. First, the multivariable model was repeated using the total-score definition of inadequate bowel preparation, defined as total BBPS ≤ 5, while retaining the same covariate set as in the main model. Second, an exploratory expanded model additionally adjusted for BMI and HbA1c, which were considered clinically relevant metabolic characteristics in patients with diabetes. This expanded model was interpreted cautiously because of the reduced complete-case sample size.

To assess potential temporal or ascertainment differences between the retrospective and prospective components of the ambispective cohort, the two components were compared with respect to the primary outcome and selected baseline characteristics. An additional sensitivity analysis was performed by adding study component (retrospective vs. prospective) as a covariate to the main multivariable logistic regression model.

All statistical tests were two-sided, and the level of statistical significance was set at *p* < 0.05. *p*-values below 0.001 were reported as *p* < 0.001.

### 2.5. Ethical Considerations

The study was conducted in accordance with the principles of the Declaration of Helsinki and the applicable legislative framework of the Republic of Croatia governing personal data protection. Given the ambispective observational design and anonymized processing of patient data, the study was approved by the Ethics Committee of the University Hospital Center Split (Class: 520-03/25-01/06; Approval No. 2181-147/01-06/LJ.Z.-25-02; approved on 31 January 2025). The requirement for individual informed consent was waived by the Ethics Committee because the study was observational and all data were analyzed in anonymized form. All data were processed exclusively for the purposes of this study.

## 3. Results

### 3.1. Sample Characteristics

A total of 194 adult patients with diabetes mellitus who underwent total colonoscopy at the Department of Gastroenterology and Hepatology, University Hospital Centre of Split, and were prepared using the institutional split-dose PEG/macrogol-based bowel preparation approach were included in the final analytic sample. All included participants met the predefined inclusion criteria.

The median age was 69 years (IQR: 12), with 137 participants (70.6%) aged ≥65 years. There were 131 men (67.5%) and 63 women (32.5%). The median body mass index (BMI), available for 143 of 194 participants (73.7%), was 28.0 kg/m^2^ (IQR: 8.1), while obesity (BMI ≥ 30 kg/m^2^) was present in 51 participants (35.7% of those with available BMI data). Regarding smoking status, 117 participants (60.3%) were non-smokers, 45 (23.2%) were current smokers, and 32 (16.5%) were former smokers. The final analytic sample comprised 151 participants from the retrospective component and 43 from the prospective component. Data regarding educational level were unavailable for 179/194 participants (92.3%) and were therefore not included in the subsequent analyses (Table 1).

Type 2 diabetes mellitus was present in 179 participants (92.3%), while 15 participants (7.7%) had type 1 diabetes mellitus. The median duration of diabetes mellitus was 11 years (IQR: 15.5), ranging from 1 to 46 years. The median glycated hemoglobin (HbA1c) level was 7.1% (IQR: 1.6), with available data for 167 participants (86.1%).

The most prescribed antidiabetic treatment consisted of oral hypoglycemic agents, used by 129 participants (66.5%), whereas combination therapy with insulin and oral hypoglycemic agents, with or without a GLP-1 receptor agonist (GLP-1RA), was administered in 30 participants (15.5%).

Microvascular complications of diabetes mellitus (neuropathy, nephropathy, or retinopathy) were present in 94 participants (48.5%), while macrovascular complications (cardiovascular disease, cerebrovascular disease, or peripheral vascular disease) were observed in 62 participants (32.0%).

Detailed characteristics and diabetes-related complications of the study population are presented in Table 2.

The most prevalent comorbid conditions were cardiovascular comorbidities (*n* = 175; 90.2%) and hematological comorbidities (*n* = 58; 29.9%). The median total number of comorbidities was 2.0 (IQR: 2.0), ranging from 0 to 7. Comorbidities of the study population are presented in Table 3.

Laboratory parameters were available for most participants, although missingness was more frequent for lipid-profile variables, which were available for 157–161 of 194 participants. Because laboratory parameters were not the primary focus of the study and were not included in the main multivariable model, detailed laboratory characteristics are presented in Supplementary Table S1.

The retrospective and prospective components of the cohort were compared to assess potential temporal or ascertainment differences (Supplementary Table S2). Most examined demographic, clinical, and diabetes-related characteristics did not differ significantly between the two components. Smoking history was more frequent in the prospective component than in the retrospective component (53.5% vs. 35.8%; *p* = 0.036). Clinically inadequate bowel preparation according to the primary combined BBPS definition was also more frequent in the prospective component (21/43 [48.8%] vs. 47/151 [31.1%]; *p* = 0.032).

### 3.2. Bowel Preparation for Colonoscopy

Indications for colonoscopy were categorized as follows: symptomatic indications (hematochezia, abdominal pain, anemia; *n* = 85; 43.8%), inflammatory bowel disease (IBD) and other indications (*n* = 66; 34.0%), and colorectal cancer screening or post-polypectomy surveillance (*n* = 43; 22.2%) (Table 4).

Chronic constipation was present in 15 participants (7.7%), while the use of tricyclic antidepressants or opioids was reported in 21 participants (10.8%). Acute infection at the time of colonoscopy was documented in 36 participants (18.6%). Previous colonoscopy had been performed in 77 participants (39.7%), whereas 100 participants (51.5%) had a history of prior abdominal surgery. According to patient status, 114 participants (58.8%) underwent colonoscopy as outpatients, while 80 participants (41.2%) were hospitalized patients (Table 4).

The median total BBPS score was 6.0 (IQR: 2.0), with values ranging from 0 to 9. According to individual colonic segments, the median BBPS scores for the right colon (BBPS-RC), transverse colon (BBPS-TC), and left colon/sigmoid colon (BBPS-LC) were 2.0 (IQR: 1.0) for all three segments (Table 5).

**Figure 1:**
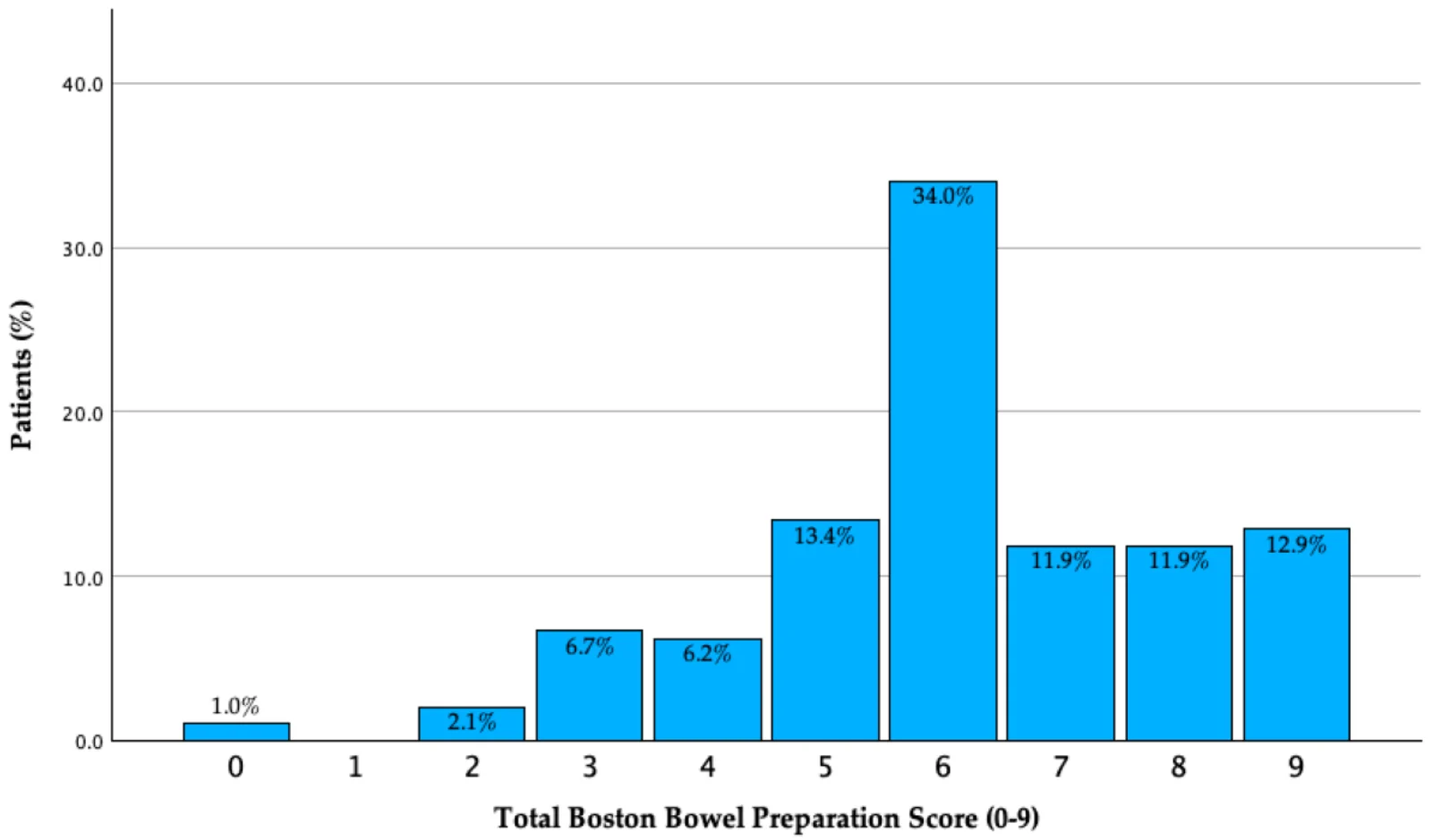
Distribution of total Boston Bowel Preparation Scale scores in the study population. The total BBPS score ranges from 0 to 9, with higher scores indicating better bowel cleansing quality (*N* = 194).

**Figure 2:**
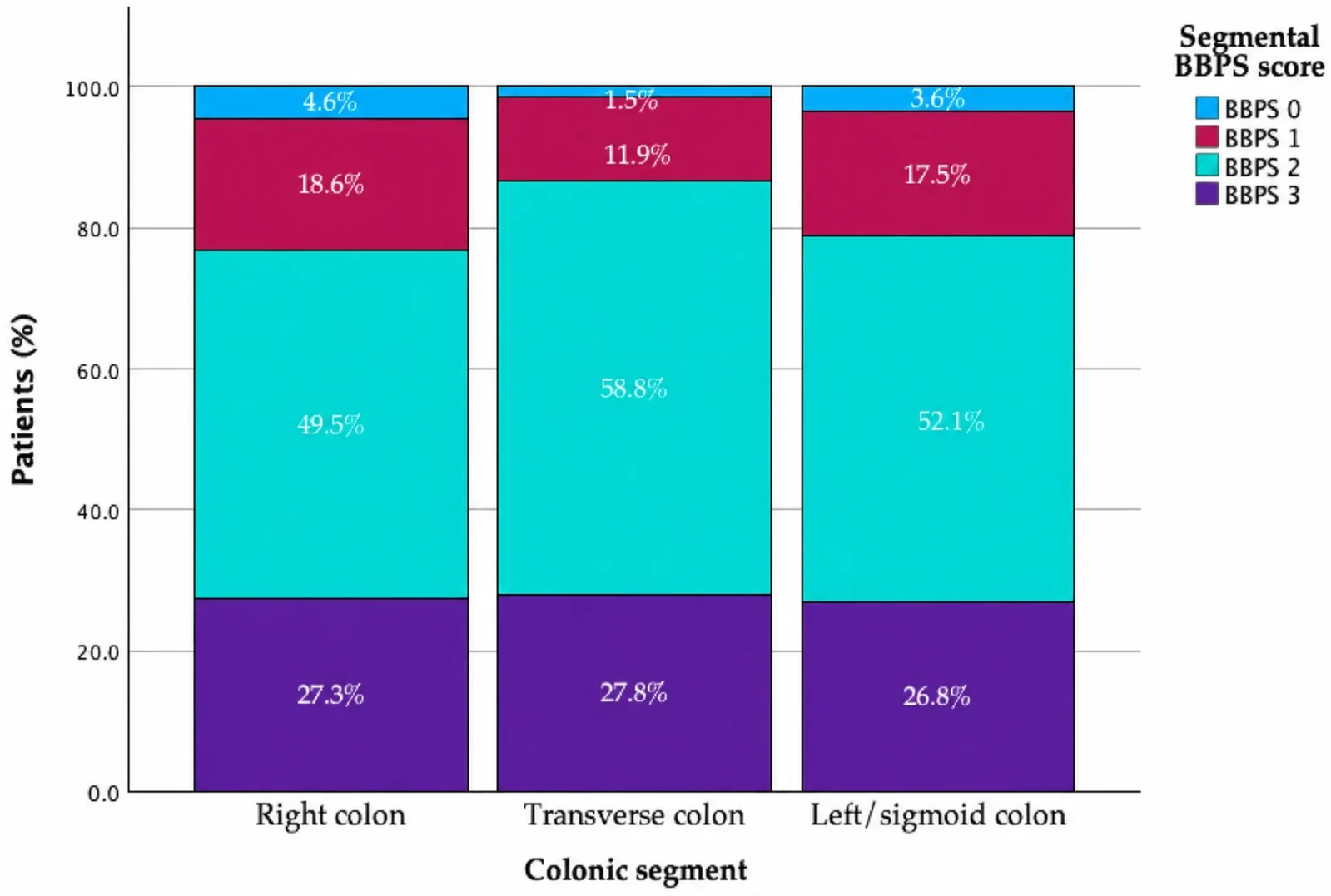
Distribution of Boston Bowel Preparation Scale scores by colonic segment. Segmental BBPS scores were assessed separately for the right colon, transverse colon and left colon/sigmoid colon. Each segment was scored from 0 to 3, with higher scores indicating better cleansing quality (*N* = 194).

Clinically inadequate bowel preparation, defined as a total BBPS score ≤ 5 or at least one segmental BBPS score ≤ 1, was identified in 68/194 participants, corresponding to a prevalence of 35.1% (95% CI: 28.3–41.8%). The remaining 126 participants (64.9%) had clinically adequate bowel preparation. In the clinically adequate bowel preparation group, the median total BBPS score was 7.0 (IQR: 2.0), whereas in the clinically inadequate bowel preparation group the median total BBPS score was 5.0 (IQR: 2.0). The distributions of total and segmental BBPS scores are presented in Figure 1 and Figure 2, respectively.

### 3.3. Bivariate Comparison According to Bowel Preparation Adequacy

For individual variables, analyses were performed using available data only, without imputation of missing values. Available sample sizes differed between analyses, primarily because BMI was available for 143/194 participants (73.7%), diabetes duration for 147/194 participants (75.8%), and HbA1c for 167/194 participants (86.1%).

A comparison of selected demographic, clinical, diabetes-related, and colonoscopy-related characteristics according to clinically inadequate bowel preparation is presented in Table 6. Additional exploratory comparisons, including individual diabetes-related complications, individual comorbidity categories, and laboratory parameters, are presented in Supplementary Table S3. Bivariate comparisons were interpreted as exploratory and were primarily used to characterize the cohort rather than to identify definitive independent predictors.

Participants with clinically inadequate bowel preparation had a longer duration of diabetes mellitus than those with clinically adequate bowel preparation (median 13.0 vs. 10.0 years; *p* = 0.037). No statistically significant differences were observed between the groups regarding age, sex, BMI, smoking history, type of diabetes mellitus, HbA1c, antidiabetic therapy, microvascular complications, macrovascular complications, chronic constipation, use of tricyclic antidepressants or opioids, outpatient status, previous colonoscopy, previous abdominal surgery, acute infection, or indication for colonoscopy.

Although diabetes duration showed a nominally significant difference in the unadjusted bivariate comparison, this association did not remain statistically significant after Benjamini–Hochberg FDR correction. Therefore, bivariate findings were interpreted as exploratory, and the association between diabetes duration and clinically inadequate bowel preparation was further evaluated using the prespecified multivariable logistic regression model.

### 3.4. Multivariable Logistic Regression

Before fitting the main multivariable model, participants included in the complete-case analysis (*n* = 147) were compared with those excluded because of missing diabetes-duration data (*n* = 47; Supplementary Table S4). The prevalence of clinically inadequate bowel preparation was similar between the two groups (35.4% vs. 34.0%; *p* = 0.868). Participants with missing diabetes-duration data were older and had a greater comorbidity burden; microvascular complications were less frequent, and acute infection was more frequent. No statistically significant differences were observed for the other examined key characteristics.

In the main multivariable logistic regression model, longer diabetes duration remained associated with clinically inadequate bowel preparation after multivariable adjustment. Each 5-year increase in diabetes duration was associated with higher odds of clinically inadequate bowel preparation after adjustment for age, sex, outpatient status, chronic constipation, and use of tricyclic antidepressants or opioids (adjusted OR 1.27, 95% CI 1.05–1.54; *p* = 0.015; *N* = 147). None of the other covariates included in the model were statistically significantly associated with clinically inadequate bowel preparation (Table 7).

The model was based on complete-case analysis and included 147 participants, of whom 52 had clinically inadequate bowel preparation. There was no evidence of violation of the linearity-in-the-logit assumption for diabetes duration (Box–Tidwell interaction *p* = 0.738) or age (*p* = 0.915). There was also no evidence of poor model fit according to the Hosmer–Lemeshow test (χ^2^ = 7.02, df = 8, *p* = 0.535), and no relevant multicollinearity was observed among the predictors (all variance inflation factors <1.13).

### 3.5. Sensitivity Analyses

To evaluate the robustness of the primary finding, sensitivity analyses were performed using an alternative outcome definition and an additional exploratory covariate-adjustment model.

First, the multivariable model was repeated using the total-score definition of inadequate bowel preparation, defined as total BBPS ≤ 5, while retaining the same covariate set as in the main model. In this model, the association between longer diabetes duration and inadequate bowel preparation remained consistent. Each 5-year increase in diabetes duration was associated with higher odds of inadequate bowel preparation (adjusted OR 1.30, 95% CI 1.06–1.59; *p* = 0.011; *N* = 147; events = 44). No other covariates were statistically significantly associated with inadequate bowel preparation in this sensitivity model.

Second, an exploratory expanded model was fitted using the primary clinically inadequate bowel preparation definition, with additional adjustment for BMI and HbA1c among participants with available data. In this model, the association between diabetes duration and clinically inadequate bowel preparation also remained statistically significant (adjusted OR 1.43, 95% CI 1.10–1.86; *p* = 0.007; *N* = 112; events = 41). Age was also statistically significant in this expanded model, whereas BMI and HbA1c were not. This model was interpreted cautiously because of the reduced complete-case sample size.

In an additional sensitivity analysis, study component (retrospective vs. prospective) was added to the main multivariable model to account for potential temporal or ascertainment differences between the two components of the ambispective cohort. The association between diabetes duration and clinically inadequate bowel preparation remained essentially unchanged (adjusted OR per 5-year increase 1.30, 95% CI 1.07–1.59; *p* = 0.009; *N* = 147; events = 52). The prospective study component was also associated with clinically inadequate bowel preparation in this model (adjusted OR 2.28, 95% CI 1.01–5.16; *p* = 0.048).

Sensitivity model diagnostics did not indicate major concerns regarding model fit or multicollinearity. Overall, the sensitivity analyses supported the robustness of the main finding that longer diabetes duration was associated with clinically inadequate bowel preparation. However, diabetes duration should not be used as a stand-alone decision rule, and the finding requires validation in larger prospective cohorts.

## 4. Discussion

### 4.1. Principal Findings

In this ambispective observational study of 194 adult patients with diabetes mellitus who underwent total colonoscopy using the institutional split-dose PEG/macrogol-based bowel preparation approach, clinically inadequate bowel preparation was observed in 35.1% of participants. Clinically inadequate bowel preparation was defined using a combined total-and-segmental BBPS criterion, namely total BBPS ≤ 5 or at least one segmental BBPS score ≤ 1. This finding indicates that more than one-third of patients with diabetes had clinically inadequate bowel cleansing despite use of the institutional split-dose PEG/macrogol-based bowel preparation approach. The use of a combined total-and-segmental BBPS definition provided a clinically more conservative estimate of inadequate preparation by accounting not only for the overall score but also for insufficient cleansing in individual colonic segments.

The main finding of the study was that longer diabetes duration remained associated with clinically inadequate bowel preparation in the prespecified multivariable logistic regression model. Specifically, each 5-year increase in diabetes duration was associated with higher odds of clinically inadequate preparation after adjustment for age, sex, outpatient status, chronic constipation, and use of tricyclic antidepressants or opioids (adjusted OR 1.27, 95% CI 1.05–1.54; *p* = 0.015).

The association between diabetes duration and inadequate bowel preparation remained consistent across sensitivity analyses. Therefore, the findings suggest that diabetes duration may be a clinically accessible marker of increased risk for inadequate bowel cleansing within diabetic populations, although further prospective validation is needed.

Importantly, the present study was not designed to re-establish diabetes mellitus itself as a risk factor for inadequate bowel preparation, as this association has already been reported in previous studies, reviews, and meta-analyses [3,6,7,8,9,10]. Rather, the study focused on risk variation within a cohort composed exclusively of patients with diabetes mellitus and explored whether routinely available clinical variables may help identify patients at higher risk of inadequate cleansing.

In this context, the findings should be interpreted as exploratory and hypothesis-generating real-world evidence rather than as definitive evidence of a causal relationship. Diabetes duration should not be used as a stand-alone decision rule for intensified bowel preparation, but it may be considered a candidate variable for future prospective risk-stratification models.

### 4.2. Interpretation in Relation to Previous Evidence

The observed frequency of clinically inadequate bowel preparation is consistent with previous evidence showing that patients with diabetes mellitus are at increased risk of suboptimal bowel cleansing [6,7,8,9,10,18]. Previous studies and meta-analyses have identified diabetes as one of several factors associated with inadequate preparation, together with older age, inpatient status, constipation, comorbidity burden, narcotic use, and incomplete adherence to bowel preparation instructions [6,7,18]. Studies focused specifically on patients with diabetes have also reported poorer bowel preparation quality compared with non-diabetic controls and have suggested that standard bowel preparation regimens may be insufficient for some patients with diabetes [8,9,19,20,21]. The present study adds to this evidence by evaluating risk variation within a diabetic cohort and showing that clinically inadequate bowel preparation remains frequent despite use of the institutional split-dose PEG/macrogol-based bowel preparation approach.

The association between longer diabetes duration and clinically inadequate bowel preparation is biologically plausible. Long-standing diabetes may be associated with autonomic dysfunction, delayed gastric emptying, impaired colonic transit, and constipation, all of which may reduce the effectiveness of standard bowel cleansing regimens [20,22]. Nevertheless, diabetes duration should be interpreted as a surrogate marker of cumulative disease burden rather than as direct evidence of diabetes-related gastrointestinal dysmotility. Diabetic autonomic neuropathy and objective autonomic function testing, gastroparesis, objective gastrointestinal transit, longitudinal glycemic exposure, glycemic variability, hypoglycemia during preparation, and detailed medication adherence were not systematically assessed in the present study. Therefore, the proposed mechanism remains plausible but was not directly demonstrated by our data.

HbA1c was not significantly associated with clinically inadequate bowel preparation in the bivariate analysis, and additional adjustment for BMI and HbA1c did not eliminate the association between diabetes duration and the primary outcome. This may suggest that diabetes duration captures aspects of long-term disease burden that are not fully reflected by a single recent measure of glycemic control. However, this interpretation remains limited because longitudinal glycemic exposure, glycemic variability, hypoglycemia during bowel preparation, and medication adherence were not systematically available. These factors should be evaluated in future prospective studies.

In contrast to some previous reports, age, sex, BMI, diabetes type, antidiabetic therapy, chronic constipation, and use of tricyclic antidepressants or opioids were not identified as robustly associated with clinically inadequate bowel preparation in the present analysis [7,23]. Microvascular and macrovascular complications also did not differ significantly between clinically adequate and clinically inadequate preparation groups in the bivariate comparison. These findings may reflect the modest sample size, missing data for some variables, possible underreporting of constipation or autonomic symptoms, and the relatively homogeneous nature of a cohort composed entirely of patients with diabetes mellitus.

### 4.3. Clinical Implications

Clinically inadequate bowel preparation has important implications because it may impair mucosal visualization, reduce lesion detection, prolong procedure time, and increase the likelihood of repeat colonoscopy [1,2,4]. Although adenoma detection, missed lesions, repeat procedures, and colorectal cancer outcomes were not directly evaluated in the present study, the high frequency of clinically inadequate preparation observed in this diabetic cohort supports the need for improved pre-procedural assessment and preparation optimization in this population.

Current bowel preparation guidelines emphasize split-dose regimens, dietary modification, adequate patient instructions, and appropriate timing of bowel cleansing agents, but diabetes-specific recommendations remain limited [13,14,15,24]. In patients with diabetes, preparation planning may need to account for glucose monitoring, hydration, renal function, gastrointestinal dysmotility, and temporary adjustment of antihyperglycemic therapy [5,11,12]. These issues are increasingly relevant with the use of newer glucose-lowering medications, including GLP-1 receptor agonists and SGLT2 inhibitors, which may affect gastrointestinal motility, hydration status, and periprocedural metabolic risk [25,26,27,28,29,30,31,32].

The present study was not designed to evaluate specific antidiabetic drug classes, medication-withholding protocols, or periprocedural glycemic events. These factors should be systematically recorded in future prospective studies.

From a practical perspective, the present findings support further evaluation of individualized bowel preparation strategies for patients with diabetes mellitus. Such strategies could incorporate pre-procedural risk assessment based on diabetes duration together with other clinically relevant factors, including chronic constipation, suspected autonomic dysfunction, renal impairment, comorbidity burden, medication profile, adherence barriers, and previous bowel preparation quality. For patients with long-standing diabetes or additional risk factors, future studies should evaluate whether reinforced written and verbal instructions, confirmation of patient understanding, individualized hydration advice, careful adjustment of antihyperglycemic therapy, or modified dietary and bowel preparation protocols improve bowel cleansing quality. In patients with visual, sensory, or cognitive barriers, simplified instructions and involvement of caregivers may also warrant evaluation. However, these approaches remain hypothesis-generating and require prospective validation before routine implementation.

### 4.4. Strengths and Limitations

A strength of the present study is its focus on a clinically important real-world population of patients with diabetes mellitus undergoing colonoscopy. In contrast to studies that evaluate diabetes only as one of several predictors in mixed populations, the present study examined risk variation within a cohort composed exclusively of patients with diabetes. The use of the BBPS provided a standardized measure of bowel preparation quality [16,17], and the primary outcome incorporated both total and segmental BBPS scores to better reflect clinically relevant bowel cleansing adequacy. The analysis was strengthened by the use of a prespecified parsimonious multivariable model, sensitivity analyses, false-discovery-rate correction for multiple exploratory bivariate comparisons, and explicit reporting of missing data and model diagnostics.

Several limitations should also be acknowledged. First, this was a single-center observational study conducted in a tertiary hospital setting with a modest sample size. The findings may therefore not be directly generalizable to other healthcare systems, outpatient-only populations, centers using different bowel preparation regimens, or settings with different patient education strategies. Incomplete colonoscopies were excluded from the analysis. Only three patients were excluded for this reason, and in all three cases the procedure was discontinued because of patient discomfort or pain rather than inadequate bowel preparation. Nevertheless, because inadequate bowel preparation may itself contribute to colonoscopy incompletion, the exclusion of incomplete procedures may have introduced some selection bias and should be considered when interpreting the observed prevalence of clinically inadequate bowel preparation.

Although adjustment for study component (retrospective vs. prospective) did not materially alter the association between diabetes duration and clinically inadequate bowel preparation, the primary outcome was more frequent in the prospective component. Therefore, temporal or ascertainment differences between the two components of the ambispective cohort cannot be excluded. In addition, chronic constipation was ascertained from documented clinical history rather than standardized diagnostic criteria such as the Rome IV criteria, which may have resulted in misclassification and limited the evaluation of its association with bowel preparation quality.

Second, residual confounding remains possible despite multivariable adjustment. Because bowel preparation was administered as part of routine clinical care and part of the cohort was collected retrospectively, detailed patient-level information on the exact commercial PEG/macrogol formulation, prescribed and actually consumed preparation volume, exact timing of dose completion, and the interval between completion of the final dose and colonoscopy could not be reliably verified for all participants. Adherence to preparation instructions, dietary compliance, hydration status, and other preparation-related factors were also not systematically available. These unmeasured or incompletely documented factors may have confounded or modified the observed association between diabetes duration and clinically inadequate bowel preparation. Usual bowel-movement frequency and the presence of colonic redundancy were also not systematically recorded and therefore could not be evaluated as potential determinants of bowel preparation quality.

Third, missing data were present for several clinically important variables, particularly BMI, diabetes duration, HbA1c, and lipid parameters. Multivariable analyses were based on complete cases, which reduced the effective sample size. Comparison of participants included in the complete-case model with those excluded because of missing diabetes-duration data showed a similar prevalence of clinically inadequate bowel preparation, but differences were observed in age, comorbidity burden, microvascular complications, and acute infection. These findings indicate that missingness was associated with some measured patient characteristics; therefore, complete-case selection bias cannot be excluded. Multiple imputation was not performed because of the exploratory design, modest sample size, and limited availability of auxiliary variables needed to support a robust imputation model. Therefore, the observed associations should be interpreted cautiously.

Fourth, BBPS scores were extracted from routine colonoscopy reports and were assigned by the performing endoscopists during routine clinical practice. Endoscopists were not formally blinded to patients’ clinical characteristics, including diabetes duration, and no centralized blinded reassessment was performed. Therefore, inter-endoscopist variability and assessment bias cannot be completely excluded.

Finally, several diabetes-specific variables were not systematically assessed. Although neuropathy was recorded as a diabetes-related complication, diabetic autonomic neuropathy was not separately identified or systematically assessed. Other unavailable or incompletely characterized factors included gastroparesis, neuropathy severity, longitudinal glycemic exposure, glycemic variability, hypoglycemia during bowel preparation, medication adherence, detailed exposure to newer antihyperglycemic agents, and objective measures of gastrointestinal transit. Objective autonomic function testing, including cardiovascular autonomic reflex testing, was not performed. Therefore, the proposed mechanism linking longer diabetes duration with impaired bowel cleansing remains biologically plausible but was not directly demonstrated by our data.

### 4.5. Future Research

Future prospective multicenter studies are needed to validate the association between diabetes duration and clinically inadequate bowel preparation in larger and more diverse diabetic populations. Such studies should systematically collect preparation-related variables, including adherence to preparation instructions, completion of the prescribed regimen, dietary compliance, hydration status, exact timing of split-dose administration, and quality of patient education.

Future research should also include more detailed diabetes-specific variables, such as autonomic neuropathy, gastroparesis, neuropathy severity, longitudinal glycemic exposure, glycemic variability, periprocedural hypoglycemia or hyperglycemia, medication adherence, and exposure to newer antihyperglycemic agents. These data would help clarify whether diabetes duration is independently associated with bowel preparation quality or primarily acts as a surrogate marker of cumulative diabetes-related burden.

Finally, randomized or well-designed prospective interventional studies should evaluate whether individualized diabetes-specific preparation strategies can improve bowel cleansing quality and clinically relevant colonoscopy outcomes. Such strategies may include reinforced patient education, optimized split-dose timing, individualized dietary and hydration advice, structured glucose monitoring, and careful adjustment of antihyperglycemic therapy. Until such evidence is available, diabetes duration should be considered only as a candidate component of future risk-stratification models, not as a stand-alone criterion for intensified bowel preparation.

## 5. Conclusions

In this single-center ambispective observational study of adult patients with diabetes mellitus undergoing total colonoscopy, clinically inadequate bowel preparation was observed in 35.1% of participants despite the use of the institutional split-dose PEG/macrogol-based bowel preparation approach.

Longer diabetes duration remained associated with clinically inadequate bowel preparation after multivariable adjustment, and this association remained consistent across sensitivity analyses. However, the findings should be interpreted cautiously given the observational design, missing data, complete-case analysis, residual confounding, and limited external validity. Diabetes duration should not be considered a stand-alone criterion for intensified bowel preparation but may represent a candidate clinical characteristic for evaluation in future prospective risk-stratification studies. These findings should therefore be regarded as incremental, hypothesis-generating evidence regarding risk variation within patients with diabetes rather than as a basis for changing clinical practice. Prospective multicenter studies and interventional trials are needed to validate these findings and to determine whether individualized diabetes-specific preparation strategies can improve bowel cleansing quality and colonoscopy outcomes.

## Figures and Tables

**Table 1:** Demographic Characteristics of the Study Population (*N* = 194).

Demographic Characteristic	Value
Age (years), Md [IQR]	69.0 [12.0]
<50 years	6 (3.1%)
50–64 years	51 (26.3%)
≥65 years	137 (70.6%)
Male sex, *n* (%)	131 (67.5%)
BMI (kg/m^2^), Md [IQR] ^a^	28.0 [8.1]
<25 kg/m^2^	42 (29.4%)
25–29.9 kg/m^2^	50 (35.0%)
≥30 kg/m^2^	51 (35.7%)
Smoking status, *n* (%)	
Current smoker	45 (23.2%)
Non-smoker	117 (60.3%)
Former smoker	32 (16.5%)

**Abbreviations**: Md = median; IQR = interquartile range (Q75–Q25); BMI = body mass index. ^a^ BMI data were available for 143/194 participants (73.7%); percentages for BMI categories were calculated based on the number of participants with available BMI data.

**Table 2:** Characteristics and Diabetes-Related Complications of the Study Population (*N* = 194).

Diabetes Mellitus Characteristics	Value
Type 2 diabetes mellitus, *n* (%)	179 (92.3%)
Duration of diabetes mellitus (years), Md [IQR] ^a^	11 [15.5]
HbA1c (%), Md [IQR] ^b^	7.1 [1.6]
Antidiabetic therapy, *n* (%)	
No therapy	11 (5.7%)
Insulin therapy	24 (12.4%)
Oral hypoglycemic agents	129 (66.5%)
Insulin + oral hypoglycemic agents ± GLP-1RA	30 (15.5%)
**Diabetes-Related Complications**	
Microvascular complications (≥1), *n* (%)	94 (48.5%)
Neuropathy	35 (18.0%)
Nephropathy	60 (30.9%)
Retinopathy	19 (9.8%)
Macrovascular complications (≥1), *n* (%)	62 (32.0%)
Cardiovascular complications	40 (20.6%)
Cerebrovascular complications	12 (6.2%)
Peripheral vascular disease	29 (14.9%)

**Abbreviations**: Md = median; IQR = interquartile range; HbA1c = glycated hemoglobin; GLP-1RA = glucagon-like peptide-1 receptor agonist. ^a^ Duration of diabetes mellitus was available for 147/194 participants (75.8%). ^b^ HbA1c data were available for 167/194 participants (86.1%).

**Table 3:** Comorbidities of the Study Population (*N* = 194).

Comorbidities; *n* (%)	
Cardiovascular	175 (90.2%)
Gastrointestinal	52 (26.8%)
Hematological	58 (29.9%)
Endocrine	31 (16.0%)
Nephrological	24 (12.4%)
Urological/Gynecological	25 (12.9%)
Neurological/Ophthalmological	12 (6.2%)
Pulmonary	22 (11.3%)
Psychiatric	20 (10.3%)
Musculoskeletal	31 (16.0%)
Oncological	18 (9.3%)
Rheumatological	8 (4.1%)
Total number of comorbidities, Md [IQR]	2.0 [2.0]

**Abbreviations**: Md = median; IQR = interquartile range.

**Table 4:** Clinical and Anamnestic Characteristics of the Study Population (*N* = 194).

Clinical and Anamnestic Characteristics; *n* (%)	
Indication for colonoscopy	
Screening/surveillance	43 (22.2%)
Symptomatic	85 (43.8%)
IBD/other	66 (34.0%)
Chronic constipation	15 (7.7%)
Use of TCAs/opioids	21 (10.8%)
Previous abdominal surgery	100 (51.5%)
Previous colonoscopy	77 (39.7%)
Acute infection	36 (18.6%)
Outpatient status	114 (58.8%)

**Abbreviations**: TCAs = tricyclic antidepressants; IBD = inflammatory bowel disease.

**Table 5:** Bowel Preparation for Colonoscopy and Distribution of Scores According to the Boston Bowel Preparation Scale (*N* = 194).

Bowel Preparation and Bbps	Value
Total BBPS score, Md [IQR]	6.0 [2.0]
BBPS-RC, Md [IQR]	2.0 [1.0]
BBPS-TC, Md [IQR]	2.0 [1.0]
BBPS-LC, Md [IQR]	2.0 [1.0]
Clinically inadequate bowel preparation (BBPS ≤ 5 OR any segmental BBPS ≤ 1), *n* (%)	68 (35.1%)

**Abbreviations**: BBPS = Boston Bowel Preparation Scale; BBPS-RC = right colon; BBPS-TC = transverse colon; BBPS-LC = left colon/sigmoid colon.

**Table 6:** Comparison of Selected Demographic, Clinical, Diabetes-Related, and Colonoscopy-Related Characteristics According to Clinically Adequate or Inadequate Bowel Preparation (*N* = 194).

Variable	Adequate (*n* = 126)	Inadequate (*n* = 68)	*p*-Value
**Demographic Data**			
Age (years), Md [IQR]	69.5 [12.0]	69.0 [12.0]	0.634 ^a^
Male sex, *n* (%)	84 (66.7%)	47 (69.1%)	0.728 ^b^
BMI (kg/m^2^), Md [IQR]	28.1 [8.0]	27.3 [7.9]	0.454 ^a^
Smoking history, *n* (%)	49 (38.9%)	28 (41.2%)	0.756 ^b^
**Diabetes Characteristics**			
Type 2 diabetes mellitus, *n* (%)	117 (92.9%)	62 (91.2%)	0.676 ^b^
Duration of diabetes mellitus (years), Md [IQR]	10.0 [13.5]	13.0 [14.2]	**0.037 ^a^**
HbA1c (%), Md [IQR]	7.1 [1.6]	7.2 [1.5]	0.535 ^a^
Antidiabetic therapy, *n* (%)			
No therapy	6 (4.8%)	5 (7.4%)	0.675 ^b^
Insulin therapy	15 (11.9%)	9 (13.2%)	
Oral hypoglycemic agents	83 (65.9%)	46 (67.6%)	
Insulin + oral hypoglycemic agents ± GLP-1RA	22 (17.5%)	8 (11.8%)	
**Diabetes-Related Complications**			
Microvascular complications (≥1), *n* (%)	60 (47.6%)	34 (50.0%)	0.752 ^b^
Macrovascular complications (≥1), *n* (%)	35 (27.8%)	27 (39.7%)	0.089 ^b^
**Comorbidity Burden and Clinical Data**			
Total number of comorbidities, Md [IQR]	2.0 [1.8]	3.0 [2.0]	0.955 ^a^
Chronic constipation, *n* (%)	9 (7.1%)	6 (8.8%)	0.676 ^b^
Use of TCAs/opioids, *n* (%)	17 (13.5%)	4 (5.9%)	0.104 ^b^
Previous abdominal surgery, *n* (%)	62 (49.2%)	38 (55.9%)	0.375 ^b^
Previous colonoscopy, *n* (%)	49 (38.9%)	28 (41.2%)	0.756 ^b^
Acute infection, *n* (%)	19 (15.1%)	17 (25.0%)	0.090 ^b^
Outpatient status, *n* (%)	74 (58.7%)	40 (58.8%)	0.990 ^b^
Indication for colonoscopy, *n* (%)			
Screening/surveillance	28 (22.2%)	15 (22.1%)	0.819 ^b^
Symptomatic	57 (45.2%)	28 (41.2%)	
IBD/other	41 (32.5%)	25 (36.8%)	

**Abbreviations**: BMI = body mass index; HbA1c = glycated hemoglobin; GLP-1RA = glucagon-like peptide-1 receptor agonist; TCAs = tricyclic antidepressants; IBD = inflammatory bowel disease; Md = median; IQR = interquartile range. *p*-values shown are unadjusted. Additional exploratory bivariate comparisons are provided in Supplementary Table S3. ^a^ Mann–Whitney U test; ^b^ Pearson chi-square test.

**Table 7:** Multivariable Logistic Regression Analysis of Factors Associated with Clinically Inadequate Bowel Preparation (*n* = 147).

Covariate	Adjusted OR	95% CI	*p*-Value
Duration of diabetes mellitus, per 5-year increase	1.27	1.05–1.54	0.015
Age, per 1-year increase	0.98	0.94–1.02	0.267
Female sex (ref. male)	0.82	0.39–1.74	0.610
Outpatient status (ref. inpatient)	0.88	0.43–1.82	0.728
Chronic constipation (ref. no)	1.97	0.50–7.73	0.333
Use of TCAs/opioids (ref. no)	0.54	0.15–1.87	0.328

**Abbreviations**: OR = odds ratio; CI = confidence interval; TCAs = tricyclic antidepressants. Clinically inadequate bowel preparation was defined as a total BBPS score ≤ 5 or at least one segmental BBPS score ≤ 1. The model was adjusted for all covariates shown in the table and was based on complete-case analysis (clinically inadequate bowel preparation events = 52). For categorical variables, odds ratios compare the listed category with the reference category shown in parentheses. For continuous variables, odds ratios are expressed per specified unit increase.

## Data Availability

The original contributions presented in this study are included in the article. Further inquiries can be directed to the corresponding authors.

## Supplementary Materials

The following supporting information can be downloaded at https://www.mdpi.com/article/10.3390/diagnostics16182935/s1: Table S1: Laboratory Parameters of the Study Population (*N* = 194). Table S2: Comparison of the Retrospective and Prospective Components of the Ambispective Cohort. Table S3: Additional Exploratory Bivariate Comparisons According to Clinically Adequate or Inadequate Bowel Preparation (*N* = 194). Table S4: Comparison of Participants Included in and Excluded from the Main Complete-Case Regression Analysis.

## Abbreviations

The following abbreviations are used in this manuscript:

ALT	alanine aminotransferase
AST	aspartate aminotransferase
BBPS	Boston Bowel Preparation Scale
BBPS-LC	left colon/sigmoid colon Boston Bowel Preparation Scale score
BBPS-RC	right colon Boston Bowel Preparation Scale score
BBPS-TC	transverse colon Boston Bowel Preparation Scale score
BMI	body mass index
CI	confidence interval
CRP	C-reactive protein
DM	diabetes mellitus
GLP-1RA	glucagon-like peptide-1 receptor agonist
HbA1c	glycated hemoglobin
HDL	high-density lipoprotein
IBD	inflammatory bowel disease
IQR	interquartile range
LDH	lactate dehydrogenase
LDL	low-density lipoprotein
MCV	mean corpuscular volume
PEG	polyethylene glycol
SGLT2	sodium–glucose cotransporter-2
