# Supplementary material for: Factors Associated with Clinically Inadequate Bowel Preparation Among Patients with Diabetes Mellitus Undergoing Colonoscopy: A Single-Center Observational Study
Source: Diagnostics (Basel). 2026 Sep 11;16(18):2935. doi: 10.3390/diagnostics16182935 (PMC13605215; doi:10.3390/diagnostics16182935)
Supplement: Supplementary file 1 [file diagnostics-16-02935-s001.zip › diagnostics-4489392-supplementary.pdf]

Table S1. Laboratory Parameters of the Study Population (N = 194).

Laboratory parameter	n	Md [IQR]
**COMPLETE BLOOD COUNT**		
Erythrocytes (×10^{12}/L)	191	4.52 [1.01]
Hemoglobin (g/L)	191	128.0 [33.5]
MCV (fL)	191	88.4 [7.5]
**RENAL FUNCTION**		
Urea (mmol/L)	189	6.7 [3.7]
Creatinine (μmol/L)	192	83.5 [42.0]
**LIPID PROFILE**		
Total cholesterol (mmol/L)	161	4.0 [1.4]
LDL cholesterol (mmol/L)	160	2.0 [1.13]
HDL cholesterol (mmol/L)	157	1.2 [0.50]
Triglycerides (mmol/L)	157	1.50 [0.80]
**INFLAMMATORY PARAMETERS**		
CRP (mg/L)	178	3.3 [15.2]
**LIVER ENZYMES**		
AST (U/L)	190	23.0 [12.0]
ALT (U/L)	192	23.5 [21.0]
LDH (U/L)	181	180.0 [43.0]
**ELECTROLYTES**		
Sodium (mmol/L)	192	140.0 [3.0]
Potassium (mmol/L)	189	4.3 [0.7]
Chloride (mmol/L)	180	102.0 [5.0]

Abbreviations: Md = median; IQR = interquartile range (Q75–Q25); MCV = mean corpuscular volume; CRP = C-reactive protein; AST = aspartate aminotransferase; ALT = alanine aminotransferase; LDH = lactate dehydrogenase.

Table S2. Comparison of the Retrospective and Prospective Components of the Ambispective Cohort

Variable	Retrospective (n = 151)	Prospective (n = 43)	P-value
Age (years), Md [IQR]	69.0 [12.0]	69.0 [13.5]	0.698
Male sex, n (%)	102 (67.5%)	29 (67.4%)	0.989
BMI (kg/m^2), Md [IQR]	27.4 [7.5] [a]	29.0 [9.2] [b]	0.092
Smoking history, n (%)	54 (35.8%)	23 (53.5%)	**0.036**
Type 2 diabetes mellitus, n (%)	139 (92.1%)	40 (93.0%)	1.000
Duration of diabetes mellitus (years), Md [IQR]	12.0 [15.0] [c]	10.0 [11.0] [d]	0.158
HbA1c (%), Md [IQR]	7.1 [1.4] [e]	7.4 [3.3] [f]	0.409
Microvascular complications (≥1), n (%)	77 (51.0%)	17 (39.5%)	0.185
Macrovascular complications (≥1), n (%)	49 (32.5%)	13 (30.2%)	0.783
Total number of comorbidities, Md [IQR]	3.0 [2.0]	2.0 [2.0]	0.312
Chronic constipation, n (%)	10 (6.6%)	5 (11.6%)	0.330
Use of TCAs/opioids, n (%)	18 (11.9%)	3 (7.0%)	0.577

 https://doi.org/10.3390/diagnostics16182935

Previous abdominal surgery, n (%)	78 (51.7%)	22 (51.2%)	0.955
Previous colonoscopy, n (%)	56 (37.1%)	21 (48.8%)	0.165
Acute infection, n (%)	28 (18.5%)	8 (18.6%)	0.993
Outpatient status, n (%)	91 (60.3%)	23 (53.5%)	0.426
Clinically inadequate bowel preparation, n (%)	47 (31.1%)	21 (48.8%)	**0.032**

Abbreviations: Md = median; IQR = interquartile range; BMI = body mass index; HbA1c = glycated hemoglobin; TCA = tricyclic antidepressant. Continuous variables were compared using the Mann–Whitney U test. Categorical variables were compared using Pearson's chi-square test or Fisher's exact test, as appropriate. Clinically inadequate bowel preparation was defined as total BBPS ≤5 or at least one segmental BBPS score ≤1. [a] n = 110; [b] n = 33; [c] n = 110; [d] n = 37; [e] n = 131; [f] n = 36.

Table S3. Additional Exploratory Bivariate Comparisons According to Clinically Adequate or Inadequate Bowel Preparation (N = 194).

Variable	Adequate (n = 126)	Inadequate (n = 68)	P–value
**INDIVIDUAL DIABETES-RELATED COMPLICATIONS**			
Neuropathy, n (%)	22 (17.5%)	13 (19.1%)	0.775[b]
Nephropathy, n (%)	38 (30.2%)	22 (32.4%)	0.752[b]
Retinopathy, n (%)	12 (9.5%)	7 (10.3%)	0.863[b]
Cardiovascular complications, n (%)	23 (18.3%)	17 (25.0%)	0.268[b]
Cerebrovascular complications, n (%)	8 (6.3%)	4 (5.9%)	1.000[c]
Peripheral vascular disease, n (%)	16 (12.7%)	13 (19.1%)	0.232[b]
**INDIVIDUAL COMORBIDITIES**			
Cardiovascular comorbidity, n (%)	116 (92.1%)	59 (86.8%)	0.236[b]
Gastrointestinal comorbidity, n (%)	34 (27.0%)	18 (26.5%)	0.939[b]
Hematological comorbidity, n (%)	40 (31.7%)	18 (26.5%)	0.444[b]
Endocrine comorbidity, n (%)	17 (13.5%)	14 (20.6%)	0.198[b]
Nephrological comorbidity, n (%)	14 (11.1%)	10 (14.7%)	0.468[b]
Urological/gynecological comorbidity, n (%)	17 (13.5%)	8 (11.8%)	0.732[b]
Neurological/ophthalmological comorbidity, n (%)	9 (7.1%)	3 (4.4%)	0.546[c]
Pulmonary comorbidity, n (%)	13 (10.3%)	9 (13.2%)	0.541[b]
Psychiatric comorbidity, n (%)	11 (8.7%)	9 (13.2%)	0.325[b]
Musculoskeletal comorbidity, n (%)	21 (16.7%)	10 (14.7%)	0.722[b]
Oncological comorbidity, n (%)	16 (12.7%)	2 (2.9%)	**0.025[b]**
Rheumatological comorbidity, n (%)	5 (4.0%)	3 (4.4%)	1.000[c]
**LABORATORY PARAMETERS**			
Erythrocytes ($\times 10^{12}$/L), Md [IQR]	4.5 [1.0]	4.5 [1.0]	0.613[a]
Hemoglobin (g/L), Md [IQR]	127.0 [33.0]	128.0 [34.8]	0.636[a]
MCV (fL), Md [IQR]	88.2 [6.8]	88.6 [8.0]	0.774[a]
Urea (mmol/L), Md [IQR]	6.7 [3.6]	6.8 [2.9]	0.422[a]
Creatinine (µmol/L), Md [IQR]	83.0 [39.2]	84.0 [45.5]	0.609[a]
Total cholesterol (mmol/L), Md [IQR]	4.0 [1.4]	3.9 [1.2]	0.205[a]
LDL cholesterol (mmol/L), Md [IQR]	2.0 [1.2]	2.0 [1.0]	0.279[a]
Triglycerides (mmol/L), Md [IQR]	1.5 [0.8]	1.4 [1.0]	0.381[a]
HDL cholesterol (mmol/L), Md [IQR]	1.2 [0.5]	1.2 [0.5]	0.289[a]
CRP (mg/L), Md [IQR]	4.3 [12.5]	2.5 [15.8]	0.404[a]
AST (U/L), Md [IQR]	23.0 [15.0]	23.0 [10.5]	0.712[a]
ALT (U/L), Md [IQR]	23.5 [23.0]	23.0 [16.2]	0.841[a]

https://doi.org/10.3390/diagnostics16182935

LDH (U/L), Md [IQR]	178.0 [40.0]	180.5 [50.2]	0.762[a]
Sodium (mmol/L), Md [IQR]	139.5 [3.0]	140.0 [3.2]	0.916[a]
Potassium (mmol/L), Md [IQR]	4.3 [0.5]	4.2 [0.8]	0.386[a]
Chloride (mmol/L), Md [IQR]	102.0 [5.0]	102.0 [5.0]	0.409[a]

Abbreviations: BBPS = Boston Bowel Preparation Scale; MCV = mean corpuscular volume; CRP = C-reactive protein; AST = aspartate aminotransferase; ALT = alanine aminotransferase; LDH = lactate dehydrogenase; Md = median; IQR = interquartile range. Clinically inadequate bowel preparation was defined as total BBPS ≤5 or at least one segmental BBPS score ≤1. P-values shown are unadjusted and should be interpreted as exploratory. [a] Mann–Whitney U test; [b] Pearson chi-square test; [c] Fisher's exact test.

Table S4. Comparison of Participants Included in and Excluded from the Main Complete-Case Regression Analysis

Variable	Included (n = 147)	Excluded (n = 47)	P-value
Age (years), Md [IQR]	68.0 [13.0]	73.0 [9.0]	**<0.001**
Male sex, n (%)	96 (65.3%)	35 (74.5%)	0.243
BMI (kg/m^2), Md [IQR]	27.9 [8.4][a]	28.9 [6.2][b]	0.958
Type 2 diabetes mellitus, n (%)	134 (91.2%)	45 (95.7%)	0.530
HbA1c (%), Md [IQR]	7.2 [1.5][c]	7.0 [1.6][d]	0.345
Microvascular complications (≥1), n (%)	78 (53.1%)	16 (34.0%)	**0.023**
Macrovascular complications (≥1), n (%)	43 (29.3%)	19 (40.4%)	0.153
Total number of comorbidities, Md [IQR]	2.0 [2.0]	3.0 [2.0]	**0.002**
Chronic constipation, n (%)	10 (6.8%)	5 (10.6%)	0.365
Use of TCAs/opioids, n (%)	16 (10.9%)	5 (10.6%)	0.962
Previous abdominal surgery, n (%)	77 (52.4%)	23 (48.9%)	0.681
Previous colonoscopy, n (%)	59 (40.1%)	18 (38.3%)	0.823
Acute infection, n (%)	22 (15.0%)	14 (29.8%)	**0.023**
Outpatient status, n (%)	92 (62.6%)	22 (46.8%)	0.056
Clinically inadequate bowel preparation, n (%)	52 (35.4%)	16 (34.0%)	0.868

Abbreviations: Md = median; IQR = interquartile range; BMI = body mass index; HbA1c = glycated hemoglobin; TCA = tricyclic antidepressant; BBPS = Boston Bowel Preparation Scale. Continuous variables were compared using the Mann–Whitney U test. Categorical variables were compared using Pearson's chi-square test or Fisher's exact test, as appropriate. Clinically inadequate bowel preparation was defined as total BBPS ≤5 or at least one segmental BBPS score ≤1. [a] n = 118; [b] n = 25; [c] n = 137; [d] n = 30.
